# Temporal Changes in Microbial Etiology and Antibiotic Resistance Profiles of Osteoarticular Infections: An 11-Year Retrospective Analysis

**DOI:** 10.7759/cureus.95572

**Published:** 2025-10-28

**Authors:** Loubna Ait Said, Fayrouz Debbagh, Mohamed Yassir Errahmani, Zakaria Elghazouani, Malaak Abouankira, Mohammed Gharmoud, Mohammed El Hassani, Hanane El Haoury, Karima Warda, Kawtar Zahlane

**Affiliations:** 1 Microbiology, Faculty of Medicine and Pharmacy, Cadi Ayyad University, Marrakech, MAR; 2 Microbiology, Ibn Tofail University Hospital, Mohammed VI University Hospital Center, Marrakech, MAR; 3 Epidemiology and Public Health, Desbrest Institute of Epidemiology and Public Health, INSERM, University of Montpellier, Montpellier, FRA; 4 Microbiology, Ibn Tofail University Hospital, Mohammed VI University Hospital Center, Marrkech, MAR; 5 Orthopedics and Traumatology, Ibn Tofail University Hospital, Mohammed VI University Hospital Center, Marrakech, MAR

**Keywords:** antimicrobial resistance, carbapenem resistance, empirical therapy, enterobacteriaceae, gram-negative bacteria, morocco, osteoarticular infections

## Abstract

Background: Osteoarticular infections (OAIs) represent a serious clinical concern. Their management is complicated by shifting microbiological landscapes and rising antimicrobial resistance. This study aims to define the long-term trends in pathogen distribution and resistance patterns to guide empirical therapy.

Methodology: An 11-year retrospective study (2013-2023) was conducted at Ibn Tofail University Hospital, affiliated with Mohammed VI University Hospital in Marrakech, Morocco. Bacterial isolates from deep osteoarticular samples were identified using standard methods. Antimicrobial susceptibility testing was performed according to the European Committee on Antimicrobial Susceptibility Testing (EUCAST) guidelines. Statistical analysis was applied to determine significant trends over time.

Results: Among 813 bacterial isolates, Gram-negative bacilli predominated (574/813, 70.6%), followed by Gram-positive cocci (239/813, 29.4%). *Enterobacteriaceae* were the most prevalent pathogens (415/813, 51.05%), with *Enterobacter* spp. as a major representative, showing an inverse epidemiological trend with *Staphylococci*. A significant decline in isolates occurred during the COVID-19 pandemic. *Enterobacteriaceae* exhibited statistically significant increasing resistance to amoxicillin-clavulanate (21/37, 56.75% → 17/25, 68%), third-generation cephalosporins (10/37, 27% → 9/25, 36%), ertapenem (4/37, 10% → 4/25, 16%), gentamicin (7/37, 18.91% → 8/25, 32%), and ciprofloxacin (15/37, 40.54% → 13/25, 52%). No colistin resistance was detected. Extended-spectrum beta-lactamase (ESBL)-producing *Enterobacteriaceae* peaked at 35/82 (42.6%) in 2016, while carbapenem-resistant *Enterobacteriaceae *(CRE) emerged as a critical concern, rising to 3/25 (12%) in 2023. *Acinetobacter baumannii *demonstrated high but fluctuating imipenem resistance. Methicillin-resistant *Staphylococcus aureus* (MRSA) incidence was generally low but with a significant peak in 2017; all staphylococcal isolates remained susceptible to glycopeptides.

Conclusions: This retrospective longitudinal study identified *Enterobacteriaceae* as the predominant pathogens in OAIs, coupled with a significant rise in antimicrobial resistance. These findings highlight the critical need to adapt prophylactic and empirical treatment guidelines to contemporary resistance trends to ensure optimal patient outcomes.

## Introduction

Osteoarticular infections (OAIs) represent a substantial and prevalent concern, presenting challenges for both individual care and public health [1]. The diagnosis and treatment of these infections pose significant challenges, frequently necessitating prolonged hospitalizations, substantial treatment costs (particularly for prosthetic surgeries or osteosynthesis), and potential compromise to functional and, in severe cases, life prognosis [2,3]. In light of the challenges associated with treating these infections, it is imperative to attempt to confirm the infection prior to the initiation of any therapeutic interventions [4]. However, in certain emergencies, empirical antibiotic therapy may be necessary. This approach is particularly indicated in cases such as acute bacterial arthritis, acute spondylodiscitis, or animal bites likely to damage joints [5]. For patients requiring surgical interventions such as debridement, lavage/irrigation, implant-preserving synovectomy, or one- or two-stage revision, both perioperative prophylaxis and postoperative empirical therapy require broad-spectrum coverage [6]. Nevertheless, the indiscriminate utilization of antibiotics carries the potential to amplify resistance and disrupt the bacterial ecology within healthcare facilities [7]. 

The effective management of this issue, the prevention of the spread of antibiotic-resistant bacteria, and the reduction of healthcare costs are contingent upon the implementation of epidemiological surveillance. However, there is a paucity of adequate regional-level studies assessing the microbial causes and antibiotic resistance patterns of OAIs and their changes over time. This deficiency complicates the optimization of treatment protocols.

The primary objective of this study was to describe the temporal evolution and distribution of bacterial pathogens involved in OAIs at our facility over 11 years. The secondary objective was to analyze concomitant changes in their antimicrobial resistance profiles to provide an evidence base for discussing potential adaptations to empirical antibiotic therapy. 

## Materials and methods

Study design and data collection

We conducted an 11-year retrospective longitudinal study at Ibn Tofail University Hospital, affiliated with Mohammed VI University Hospital in Marrakech, Morocco. Data were extracted from the registries of the bacteriology department, and their quality was verified to ensure completeness and reliability. The study period was precisely defined, spanning from January 2013 to December 2023, in order to allow a representative and temporally bounded analysis of microbiological data. This study was restricted to adult patients (aged 18 years and older). All patients managed in the trauma-orthopedic department during this period were retrospectively identified, excluding those diagnosed with spondylodiscitis or incomplete microbiological data.

Antibiotic therapy

In our institution, all patients received single-dose preoperative prophylaxis with amoxicillin-clavulanate (AMC), extended to 48 hours postoperatively in high-risk cases (e.g., open fractures, immunosuppression). Empirical antibiotic treatment adhered strictly to contemporaneous SPILF (French Infectious Diseases Society) guidelines throughout the study period [8].

Therapy was initiated immediately after surgery and adjusted based on intraoperative microbiological results. All therapeutic decisions were made through multidisciplinary consensus (orthopedic surgeons, infectious disease specialists, and microbiologists), tailored to individual patient characteristics.

Conventional microbiological methods

Pus samples and intraoperative specimens (bone, soft tissue, joint biopsy, and/or synovial fluid) were collected either preoperatively or intraoperatively. These specimens were cultured on enriched media (Chocolate agar and blood agar), selective media (MacConkey and Chapman agar), and enrichment media (Brain Heart Infusion broth). They were incubated under standard aerobic conditions at 35-37 °C for 24-48 hours, with daily inspection for growth.

Cultures were considered positive when the organism was isolated from at least two independent samples, or from a single sample in instances when the pathogen was highly virulent (e.g., bacteria well known as causing osteoarticular infections). Semi-quantitative estimation of bacterial growth was made (scanty, moderate, abundant) as evidence for interpreting results, but was not included in the final report.

In cases of polymicrobial infections, each bacterial isolate was recorded separately to accurately represent the microbial diversity involved in osteoarticular infections. However, repeated isolates from the same patient and infection episode were excluded.

Bacterial identification was performed using classical microbiological techniques, including Gram staining from colonies, motility testing, and detection of oxidase, catalase, and coagulase (for staphylococci), complemented by a biochemical identification panel (API bioMérieux, France).

Antibiotic susceptibility testing and interpretation were conducted in accordance with the guidelines of the European Committee on Antimicrobial Susceptibility Testing (EUCAST).

Interpretation of microbiological culture results

Microbial culture results were retrospectively retrieved from patient records, with duplicate entries excluded. Multi-drug resistance (MDR) was determined based on available antimicrobial susceptibility data for five major bacterial groups (*Staphylococcus aureus*, *Enterococcus *spp., *Enterobacteriaceae*, *Acinetobacter *spp., and *Pseudomonas* spp.) according to the criteria established by the European Society of Clinical Microbiology and Infectious Diseases [9]. 

Statistical analysis

Descriptive variables were presented as frequencies and percentages to illustrate the distribution of bacterial species and trends in antimicrobial resistance over time. Group comparisons were performed using the chi-square test or Kruskal-Wallis test, depending on the nature and distribution of the data. A *P*-value < 0.05 was considered statistically significant. All statistical analyses were conducted using R software, version 4.4.1 (R Foundation for Statistical Computing, Vienna, Austria).

## Results

Type and prevalence of pathogens over time

During the 11-year study period (2013-2023), a total of 813 bacterial strains were isolated, predominantly from deep osteoarticular samples. Among them, 574/813 (70.6%) were Gram-negative bacilli and 239/813 (29.4%) were Gram-positive cocci.

**Figure 1:**
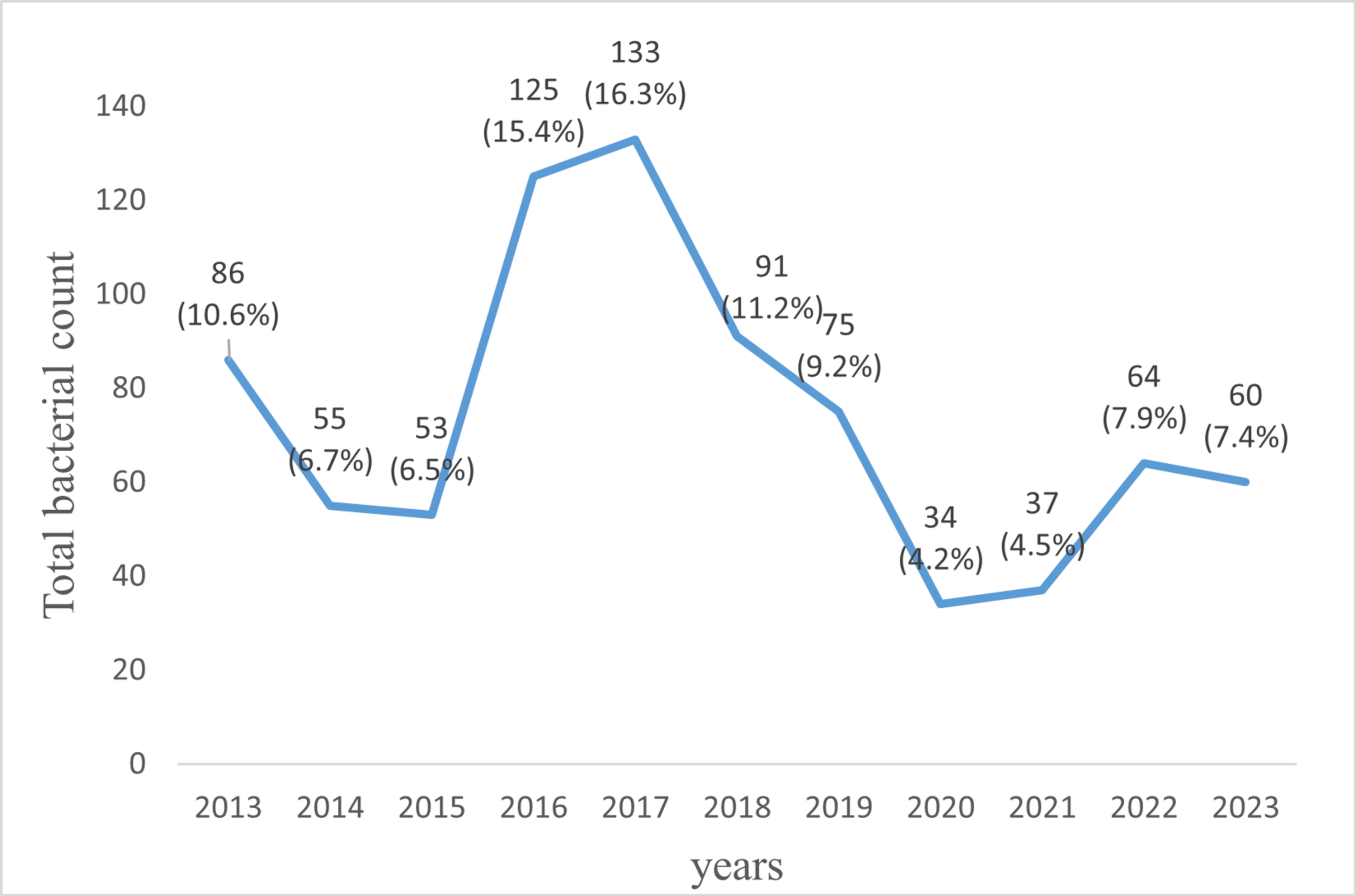
Prevalence and distribution of bacterial isolates during the study period. Data are presented as the number of isolates (n) and the corresponding percentage of the total isolates (%). Total isolates = 813.

The annual number of isolates fluctuated; it declined from 86/813 (10.6%) in 2013 to 53/813 (6.5%) in 2015 and then peaked at 133/813 (16.3%) in 2017. A significant decrease occurred during the COVID-19 pandemic, with only 34/813 (4.2%) in 2020. There was a partial recovery in 2023, with 60/813 (7.4%) (Figure 1).

In an 11-year study of osteoarticular infections, *Enterobacteriaceae* were dominant (415/813, 51%), exhibiting consistent prevalence with notable peaks in 2016-2017 and 2020-2021. Meanwhile, *Pseudomonas *spp. (88/813, 10.82%) and *Acinetobacter baumannii* (71/813, 8.73%) showed fluctuating detection rates. Gram-positive pathogens included *Staphylococcus *spp. (183/813, 22.51% overall), with *S. aureus *accounting for 129/813 (15.86%). Prevalence showed significant temporal variation (*P* < 0.001), declining sharply in 2019-2020 before rebounding to 12/37 (35.29%) in 2021.

*Enterococcus* (35/813, 4.31%) and *Streptococcus *spp*. *(21/813, 2.58%) were intermittently detected.

Strikingly, an inverse trend emerged between *Staphylococcus *and *Enterobacteriaceae*: sharp declines in *Staphylococci* coincided with surges in *Enterobacteriaceae *in 2016 and 2020 (Table 1). 

**Table 1:** Distribution of bacterial species isolated between 2013 and 2023 in osteoarticular samples. Data are presented as the number of positive isolates/total number of isolates tested (n/N), along with the corresponding percentage prevalence (%). The significance of the observed trend was assessed using Fisher's exact test. The resulting Fisher's coefficient and p-value are reported; a *P*-value < 0.05 was considered statistically significant.

Germ	2013	2014	2015	2016	2017	2018	2019	2020	2021	2022	2023	Total	Fisher's coefficient	*P*-value
*Enterobacteria *spp.	43.02% 37/86	43.64% 24/55	47.17% 25/53	65.60% 82/125	57.14% 76/133	37.36% 34/91	53.33% 40/75	73.53% 25/34	54.05% 20/37	42.19% 27/64	41.67% 25/60	51.05% 415/813	0.012	<0.001
Acinetobacter baumannii	5.81% 5/86	7.27% 4/55	15.09% 8/53	8% 10/125	10.53% 14/133	10.99% 10/91	9.33% 7/75	8.82% 3/34	0	14.06% 9/64	1.67% 1/60	8.73% 71/813	0.009	<0.001
*Pseudomonas *spp.	12.79% 11/86	16.36% 9/55	11.32% 6/53	12% 15/125	8.27% 11/133	6.59% 6/91	14.67% 11/75	5.88% 2/34	5.41% 2/37	7.81% 5/64	16.67% 10/60	10.82% 88/813	0.025	0.03
*Staphylococcus *spp.	30.23% 26/86	20% 11/55	22.64% 12/53	12.80% 16/125	23.31% 31/133	27.47% 25/91	12% 9/75	8.82% 3/34	35.29% 12/37	25% 16/64	33.33% 20/60	22.51% 183/813	0.008	<0.001
Staphylococcus aureus	23,25% 20/86	16.36% 9/55	16.98% 9/53	9.6% 12/125	19.54% 26/133	19.78% 18/91	4% 3/75	5.88% 2/34	24.32% 9/37	15.64% 10/64	18.33% 11/60	15.86% 129/813	0.020	<0.001
*Enterococcus *spp.	5.81% 5/86	5.45% 3/55	1.89% 1/53	0	0.75% 1/133	10.99% 10/91	5.33% 4/75	2.94% 1/34	5.41% 2/37	7.81% 5/64	5% 3/60	4.31% 35/813	0.007	<0.001
*Streptococcus *spp.	2.33% 2/86	7.27% 4/55	1.89% 1/53	1.60% 2/125	0	6.59% 6/91	5.33% 4/75	0	0	1.56% 1/64	1.67% 1/60	2.58% 21/813	0.100	0.52

**Figure 2:**
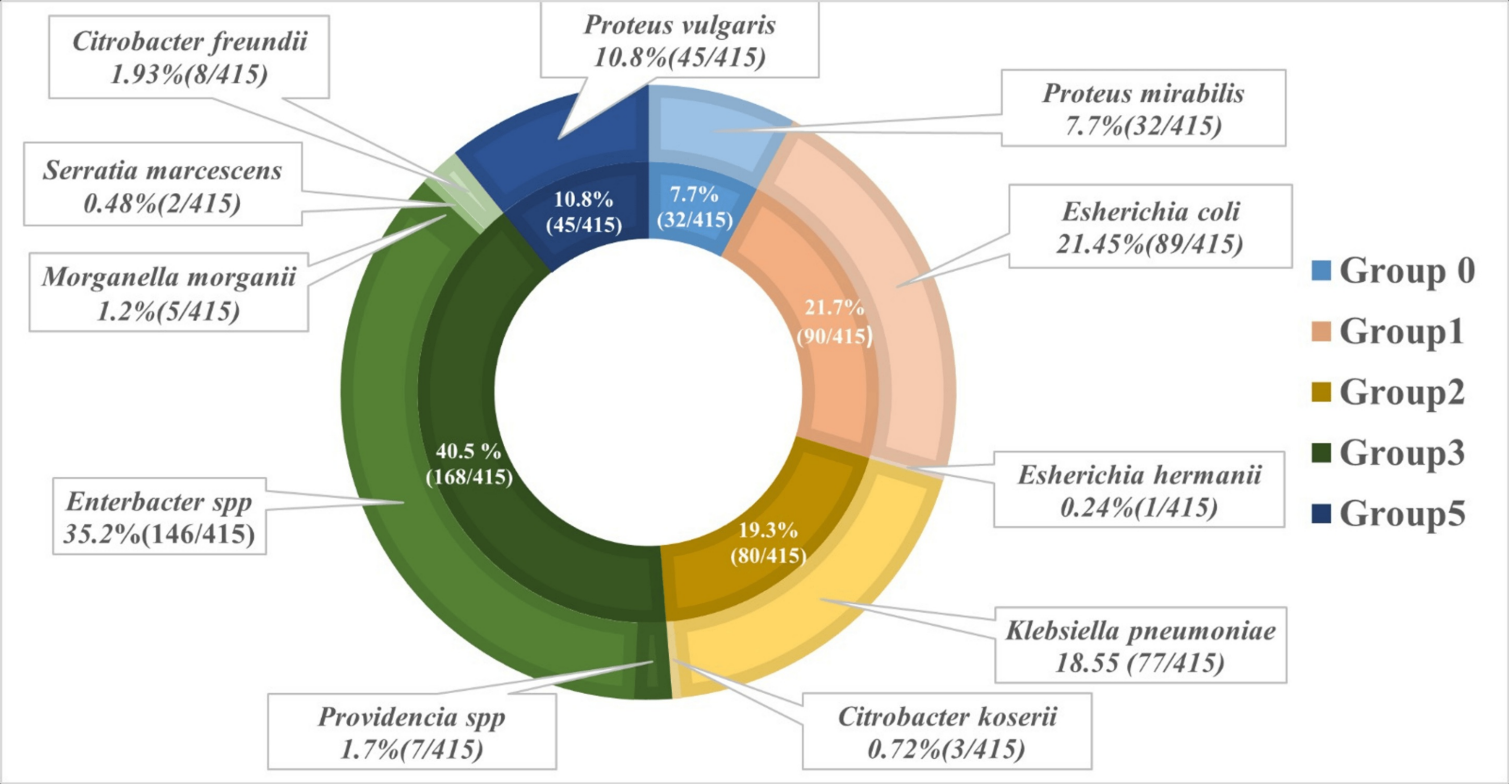
Enterobacteriaceae diversity in osteoarticular infections: groups and key species. Data are presented as the number of isolates per species/group (*n*/*N*), along with the corresponding percentage prevalence (%). Total Enterobacteriaceae isolates = 415.

Over 11 years, the epidemiological study of osteoarticular infections revealed that the group 3 bacteria, according to the *Enterobacteriaceae* natural resistance classification [10], were the most predominant (168/415, 40.5%), mainly represented by *Enterobacter *spp*.* Group 1 followed with 90/415 (21.7%), largely composed of *Escherichia coli*, while group 2 accounted for 80/415 (19.3%), predominantly represented by *Klebsiella* strains (Figure 2).

Antibiotic resistance in the most isolated bacteria

**Figure 3:**
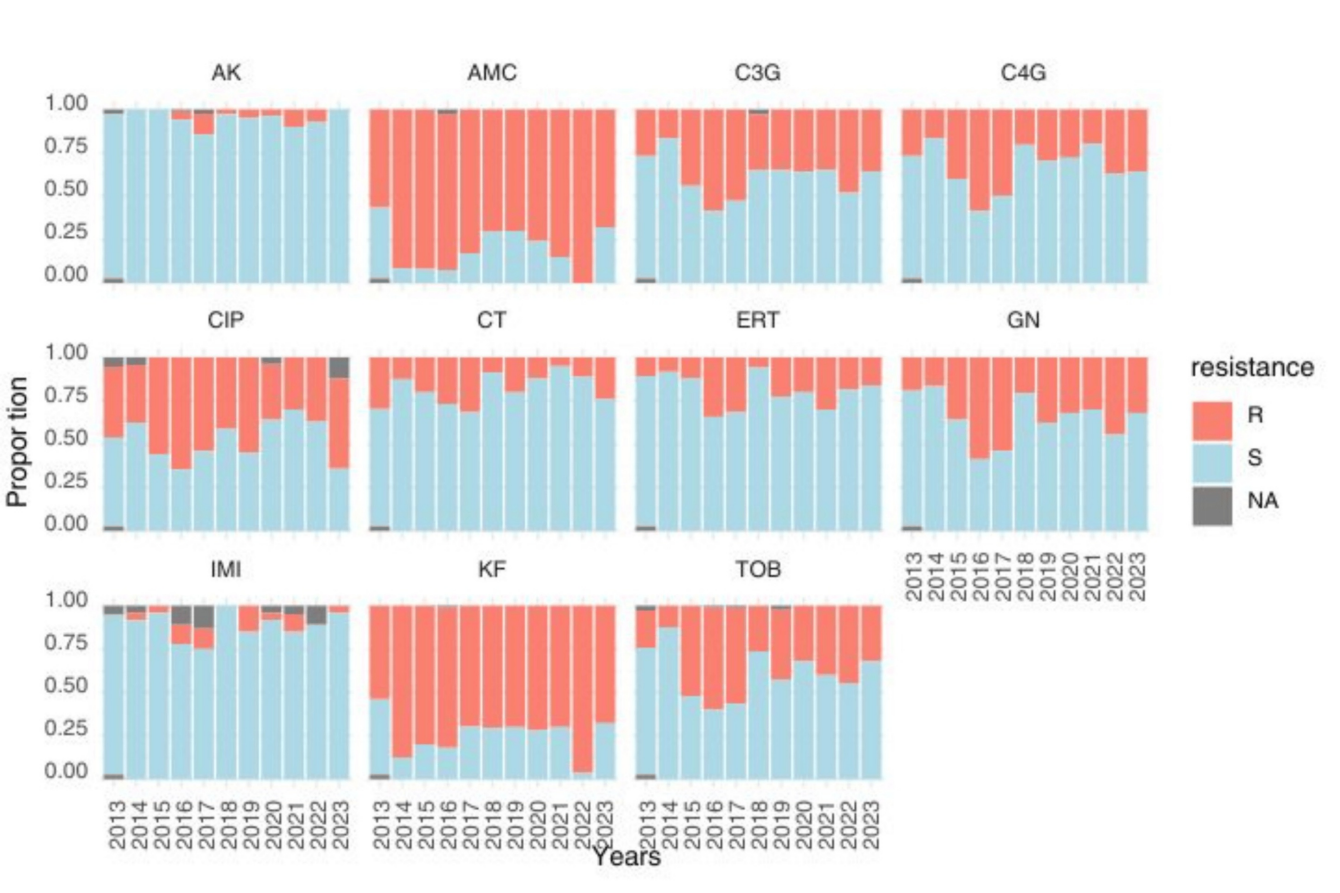
Antibiotic resistance trends of Enterobacteriaceae species isolated between 2013 and 2023. AMC, amoxicillin-clavulanate; C3G, third-generation cephalosporins; C4G, cefepime; Cip, ciprofloxacin; CT, colistin; Ert, ertapenem; Gen, gentamicin; AK, amikacin; Imi, imipenem; KF, cefalotin; Tob, tobramycin; R, resistant; S, susceptible; NA, data unavailable

During the study, resistance rates among *Enterobacteriaceae* (the most isolated Gram-negative bacteria) were monitored. Statistically significant increases in resistance were monitored for AMC, third-generation cephalosporins (Cephalosporin3G), Ertapenem, Gentamicin, Tobramycin, and Ciprofloxacin, rising from 21/37 (56.75%), 10/37 (27.02%), 4/37 (10.81%), 7/37 (18.91%), 8/37 (21.62%), and 15/37 (40.54%) in 2013 to 17/25 (68%), 9/25 (36%), 4/25 (16%), 8/25 (32%), 8/25 (32%), and 13/25 (52%) in 2023, respectively. Overall, the resistance rates for these antibiotics were 335/415 (80.72%), 176/415 (42.41%), 92/415 (22.17%), 165/415 (39.76%), 175/415 (42.17%), and 204/415 (49.16%), respectively. In contrast, no resistance to Colistin was detected at any point during the study (Table 2; Figure 3).

**Table 2:** Evolution of antibiotic resistance in Enterobacteriaceae isolated from osteoarticular infections during the study period. Data are presented as the number of positive isolates/total number of isolates tested (*n*/*N*), along with the corresponding percentage prevalence (%). The significance of the observed trend was assessed using Fisher's exact test. The resulting Fisher's coefficient and *P*-value are reported; a *P*-value less than 0.05 was considered statistically significant. Cephalosporin3G, third-generation cephalosporins

Antibiotics	2013	2014	2015	2016	2017	2018	2019	2020	2021	2022	2023	Total percentage	Fisher's coefficient	*P*-value
Amoxicillin-clavulanate	56.75% 21/37	91.66% 22/24	92% 23/25	90.24% 74/82	82.89% 63/76	70.58% 24/34	70% 28/40	76% 19/25	85% 17/20	100% 27/27	68% 17/25	80.72% 335/415	0.005	<0.001
Cefalotin	54.05% 20/37	87.5% 21/24	80% 20/25	80.48% 66/82	69.73% 53/76	70.58% 24/34	70% 28/40	72% 18/25	70% 14/20	96.29% 26/27	68% 17/25	87.46% 307/415	0.007	0.021
Cephalosporin3G	27.02% 10/37	16.66% 4/24	44% 11/25	58.53% 48/82	52.63% 40/76	32.35% 11/76	35% 14/40	36% 9/25	35% 7/20	48.14% 13/27	36% 9/25	42.41% 176/415	0.004	0.007
Cefepime	27.02% 10/37	16.67% 4/24	40% 10/25	58.54% 48/82	50% 38/76	20.59% 7/34	30% 12/40	28% 7/25	20% 4/20	37.04% 10/27	36% 9/25	38.31% 159/415	0.006	<0.01
Ertapenem	10.81% 4/37	8.33% 2/24	12% 3/25	34.14% 28/82	31.57% 24/76	5.88% 2/34	22.5% 9/40	20% 5/25	30% 6/20	18.51% 5/27	16% 4/25	22.17% 92/415	0.008	0.007
Imipenem	0	4.16% 1/24	4% 1/25	10.97% 9/82	11.84% 9/76	0	15% 6/40	4% 1/25	10% 2/20	0	4% 1/25	7.23% 30/415	0.009	0.004
Gentamicin	18.91% 7/37	16.66% 4/24	36% 9/25	58.53% 48/82	53.94% 41/76	20.58% 7/34	37.5% 15/40	32% 8/25	30% 6/20	44.44% 12/27	32% 8/25	39.76% 165/415	0.006	<0.01
Tobramycin	21.62% 8/37	12.5% 3/24	52% 13/25	58.53% 48/82	55.26% 42/76	26.47% 9/34	40% 16/40	32% 8/25	40% 8/20	44.44% 12/27	32% 8/25	42.17% 175/415	0.005	<0.001
Amikacin	0	0	0	4.88% 4/82	11.84% 9/76	2.94% 1/34	5% 2/40	4% 1/25	10% 2/20	7.41% 2/27	0	5.06% 21/415	0.030	0.09
Ciprofloxacin	40.54% 15/37	33.33% 8/24	56% 14/25	64.63% 53/82	53.94% 41/76	41.17% 14/34	55% 22/40	32% 8/25	30% 6/20	37.03% 10/27	52% 13/25	49.16% 204/415	0.020	0.037

However, resistance rates for *Pseudomonas aeruginosa* and *Acinetobacter* species remained stable throughout the study, with no significant increase observed.

**Figure 4:**
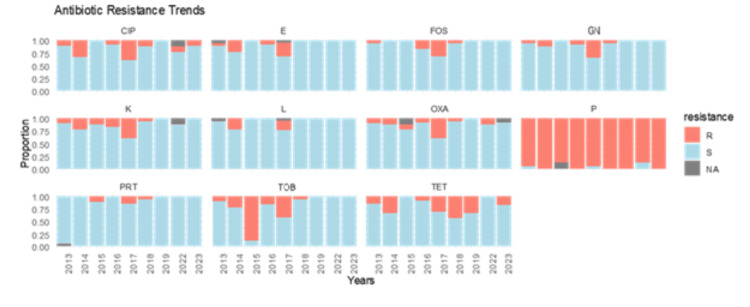
Antibiotic resistance trends of Staphylococcus aureus strains isolated between 2013 and 2023. Cip, ciprofloxacin; E, erythromycin; FOS, fosfomycin; GN, gentamicin; K, kanamycin; L, clindamycin; OXA, oxacillin; P, penicillin; PRT, pristinamycin; Tob, tobramycin; TET, tetracycline; R, resistant; S, sensible; NA, data unavailable

The resistance profile of isolated *S. aureus* strains is depicted in Table 3. Throughout the study period, methicillin resistance exhibited relative stability, with only 1-2 isolates identified annually. However, a sharp peak was observed in 2017, during which 10 resistant isolates were identified (10/26, 38.46%). Aminoglycoside resistance declined over time, dropping from 1/20 (5%) in 2013 to 0/11 (0%) in 2023 for gentamicin and from 2/20 (10%) to 0/11 (0%) for tobramycin during the same period. In contrast, fluoroquinolones resistance remained stable (*P* = 0.079), with rates of 2/20 (10%) in 2013 and 1/11 (9%) in 2023. Sensitivity to clindamycin remained high throughout the study period, except in 2014 (2/9, 22.22%) and 2017 (5/26, 19.23%) when two resistance peaks were noticed. No strain exhibited resistance to glycopeptides during the entire study period (Table 3; Figure 4).

**Table 3:** Evolution of antibiotic resistance in Staphylococcus aureus isolates from osteoarticular infections over the study period. Data are presented as the number of positive isolates/total number of isolates tested (*n*/*N*), along with the corresponding percentage prevalence (%). The significance of the observed trend was assessed using Fisher's exact test. The resulting Fisher's coefficient and *P*-value are reported; a *P*-value less than 0.05 was considered statistically significant.

Staphylococcus aureus	2013	2014	2015	2016	2017	2018	2019	2020	2021	2022	2023	Total percentage (%)	Fisher's coefficient	*P*-value
Penicillin	95% 9/20	100% 9/9	100% 9/9	100% 12/12	96.15% 25/26	100% 18/18	100% 3/3	100% 2/2	88,89% 8/9	90% 9/10	100% 11/11	96.9% 125/129	0.050	0.779
Oxacillin	10% 2/20	11.11% 1/9	11.11% 1/9	8.33% 1/12	38.46% 10/26	5.56% 1/18	0	0	11.11% 1/9	10.53% 1/10	9.09% 1/11	14.72% 19/129	0.120	0.11
Gentamicin	5% 1/20	11.11% 1/9	0	8.33% 1/12	34.61% 9/26	5.56% 1/18	0	0	0	0	0	10.07% 13/129	0.005	0.011
Tobramycin	10% 2/20	22.22% 2/9	11.11% 1/9	16.67% 2/12	42.31% 10/26	5.56% 1/18	0	0	0	0	0	13.95% 18/129	0.010	0
Kanamycin	10% 2/20	22.22% 2/9	11.11% 1/9	16.67% 2/12	38.46% 10/26	5.56% 1/18	0	0	0	10% 1/10	0	14.73% 19/129	0.070	0.074
Ciprofloxacin	10% 2/20	33.33% 9/9	0 9/9	8.33% 1/12	38.46% 12/26	11.11% 18/18	0	0	33.33% 3/9	30% 3/10	9.09% 1/11	44.96% 58/129	0.080	0.079
Erythromycin	5% 1/20	22.22% 2/9	0	8.33% 1/12	26.92% 7/26	0	0	0	33.33% 3/9	0	0	10.85% 14/129	0.020	0.027
Clindamycin	0	22.22% 2/9	0	0	19.23% 5/26	0	0	0	0	0	0	5.43% 7/129	0.030	0.039
Pristinamycin	0	0	11.11% 1/9	0	15.38% 4/26	5.56% 1/18	0	0	0	0	0	4.65% 6/129	0.100	0.299
Fosfomycin	5% 1/20	0	0	16.67% 2/12	30.77% 8/26	5.56% 1/18	0	0	0	0	0	9.30% 12/129	0.040	0.021
Tetracycline	15% 3/20	33.33% 3/9	0	8.33% 1/12	30.77% 8/26	44.44% 1/18	33.33% 1/3	0	33.33% 3/9	0	18.18% 2/11	17.05% 22/129	0.060	0.078

Changes in multi-drug-resistant bacteria over time

*Enterobacteriaceae* producing extended-spectrum beta-lactamases (ESBLs) exhibited significant variability, peaking at a prevalence of 35/82 (42.6%) in 2016, declining to 2/20 (10%) by 2021, and subsequently showing a resurgence in later years.

Carbapenem-resistant *Enterobacteriaceae *(CRE) isolates increased markedly during the study period, reaching a prevalence of 3/25 (12%) in 2023, with a notable peak observed in 2021 (3/20, 15%).

In contrast, the data consistently showed high imipenem resistance in *A. baumannii* (3/4-6/7, 75%-86%) from 2013 to 2019. However, from 2021 to 2023, there were extreme fluctuations (0/1, 0%; 5/9, 55%; and 1/1, 100%). 

The distribution of multidrug-resistant *Pseudomonas aeruginosa *reveals a sporadic pattern, with resistance absent for extended periods and then suddenly emerging at moderate to high levels before disappearing again.

Methicillin-resistant *Staphylococcus aureus* (MRSA) strains persisted at a relatively stable incidence, with one to two isolates identified annually. Notably, a sharp peak was recorded in 2017 (10/26, 38.46%), followed by a complete eradication of MRSA between 2019 and 2020 (Table 4).

**Table 4:** Evolution of multi-drug-resistant bacteria. Data are presented as the number of positive isolates/total number of isolates tested (*n*/*N*) and the corresponding percentage prevalence (%). The resulting Fisher's coefficient and *P*-value are reported; a *P*-value < 0.05 was considered statistically significant.

	2013	2014	2015	2016	2017	2018	2019	2020	2021	2022	2023	Prevalence (2013-2023)	Fisher's coefficient	*P*-value
ESBL-producing *Enterobacteriaceae* (ESBLE)	24% 9/37	16,6% 4/24	28.6% 8/25	42.6% 35/82	27.6% 21/76	17.6% 6/34	15% 6/40	10.7% 3/25	10% 2/20	25.9% 7/27	28% 7/25	26.02% 108/415	4.00	0.01
Carbapenem-resistant *Enterobacteriaceae* (CRE)	2.7% 1/37	4% 1/24	4% 1/25	11% 9/82	12% 9/76	9% 3/34	10% 4/40	12% 3/25	15% 3/20	11% 3/27	12% 3/25	9.64% 40/415	1.50	0.08
Imipenem-resistant *Acinetobacter baumannii* (IRAB)	80% 4/5	75% 3/4	87% 7/8	80% 8/10	79% 11/14	80% 8/10	86% 6/7	66% 2/3	0	55% 5/9	100% 1/1	56% 40/71	2.25	0.09
CAZ-resistant *Pseudomonas aeruginosa* (CRPA)	0	0	0	26.6% 4/15	18% 2/11	16.6% 1/6	0	0	0	40% 2/5	40% 4/10	8% 7/88	0.00	0.91
Carbapenem-resistant *Pseudomonas aeruginosa* (CRRPA)	0	0	0	26.6% 4/15	18% 2/11	16.6% 1/6	0	0	0	20% 1/5	20% 2/10	8% 7/88	0.00	0.42
Methicillin-resistant *Staphylococcus aureus* (MRSA)	10% 2/20	11.11% 1/9	11.11% 1/9	8.33% 1/12	38.46% 10/26	5.56% 1/18	0	0	11.11 1/9	10.53 1/10	9.09% 1/11	14.72 19/129	1.75	0.11

## Discussion

This longitudinal study (2013-2023) characterizes shifts in bacterial epidemiology among osteoarticular infections at Ibn Tofail University Hospital by analyzing antimicrobial resistance patterns in 813 clinical isolates. The initial decline in isolates, from 86/813 (10.6%) in 2013 to 53/813 (6.5%) in 2015, may reflect enhanced infection control measures and optimized antimicrobial stewardship [11,12]. The subsequent increase to 133/813 (16.3%) by 2017 may be due to increased surgical activity, evolving antimicrobial resistance patterns, or ecological shifts in microbial communities. In contrast, a notable decrease occurred during the 2020 - 2022 period of the pandemic, likely due to the reduction in elective procedures and the strengthening of hospital hygiene measures, which likely attenuated bacterial transmission [11,13]. The partial recovery to 60/813 (7.4%) by 2023 may indicate the gradual normalization of microbial transmission dynamics following pandemic-related disturbances.

* Enterobacteriaceae* were identified as the most frequent pathogens (415/813, 51%), with a notable peak between 2020 and 2021. They owe their predominance to their commensal nature in the digestive tract and their nosocomial potential. They cause urinary tract infections, bacteremia, and post-operative complications [14,15]. These complications were exacerbated during the pandemic due to hospital overload, equipment shortages, and postponed surgeries. Our results differ from those reported by Claire Duployez's study in France, which found that 52% of pathogens isolated from IOA were staphylococci, while *Enterobacteriaceae *accounted for only 20% [16]. This variation could be explained by contextual differences, such as population and methodology, or by the nature of the analyzed infections (postoperative, prosthesis joint, and hematogenous osteomyelitis) and the patients’ clinical state. Moreover, Lemaignen identified *S. aureus *as the predominant pathogen in osteoarticular infections [17]. Their study reported that 42.6% of osteoarticular infections were prosthetic joint infections, which are known to be strongly associated with *S. aureus.* In contrast, our research encompassed all osteoarticular infections, not making this distinction, which is a notable limitation. This lack of stratification prevents direct comparison because native joint infections and hematogenous osteomyelitis often have different microbiological profiles than device-related infections. As a result, part of the variation in pathogen distribution observed in our study may reflect differences in the types of cases included rather than true epidemiological shifts, which should be considered when interpreting the findings. These differences may also underlie organizational differences in healthcare systems, infection prevention and control policies, microbiologic laboratory testing and surveillance, local bacterial ecology, and community antibiotic use patterns [18,19]. These are possible factors, but causal inferences cannot be drawn from the present data, and harmonized, prospective multicountry studies are needed to determine whether the patterns are generalizable across settings and consistent over time.

Robinet et al. reported no significant change in antibiotic resistance across four orthopedic surgery departments over nine years, suggesting the effectiveness of local antimicrobial stewardship [20]. However, our study reveals a significant increase in enterobacterial resistance over the decade from 2013 to 2023, which can be attributed to several interconnected factors. The inappropriate use of antibiotics, particularly the excessive or empirical broad-spectrum use during the management of osteoarticular infections, has probably exerted strong selective pressure favoring the emergence of resistant strains [21,22]. Additionally, nosocomial transmission in a hospital environment, combined with inadequate surveillance and gaps in infection control practices, may have also played a crucial role [23,24]. On a molecular level, *Enterobacteriaceae *have a high ability to acquire resistance genes through plasmids and other mobile genetic elements, which exacerbates the problem [25]. These observations underline the importance of strengthening antibiotic stewardship programs, microbiological surveillance, and preventive measures to curb this alarming trend. Despite the predominance of *Enterobacteria* in OAIs during the 2020-2021 period of the pandemic (25/34, 73.53% - 20/37, 54.05%), the rate of ESBL has fallen (3/25, 10.7% - 2/20, 10%). This could be explained by the reduction in elective procedures and prolonged hospital stays, which means less exposure to endemic MDR. This could also be due to the strict reinforcement of hygiene measures (masks, hydroalcoholic gels), which limit cross-transmission; a change in antibiotic prescriptions (fewer carbapenems and more colistin or tigecycline); or a possible detection bias linked to the prioritization of samples for SARS-CoV-2. This trend contrasts with the overall increase in resistance, suggesting a shift toward other mechanisms, such as non-BLSE plasmid resistance or membrane impermeability. Genomic studies are needed to identify circulating clones during this period.

The prevalence of carbapenemase-producing *Enterobacteriaceae* (CPE) exhibited a marked increase from 1/37 (2.7%) in 2013 to sustained levels of 9/82 (11%) - 3/25 (12%) from 2016-2023, reflecting an alarming epidemiological shift.

In contrast, *Staphylococcus *spp. exhibits an inverse correlation with *Enterobacteriaceae*, demonstrating pronounced declines concurrent with surges in *Enterobacteriaceae *abundance. This antagonistic relationship can be explained by the selective effects of β-lactam antibiotics, such as AMC, which was used as prophylaxis in our study. AMC is more effective against Gram-positive bacteria (e.g., *staphylococci*), leading to their suppression. In contrast, Gram-negative *Enterobacteriaceae* often evade this pressure due to intrinsic resistance mechanisms, particularly the production of β-lactamases. Notably, in our study, 168/415 (40%) of isolated *Enterobacteriaceae* were AMPc producers, a class of β-lactamases that hydrolyze amoxicillin but remain unaffected by clavulanate inhibition. As a result, AMC prophylaxis selectively targets *Staphylococci *while allowing AMPc-producing *Enterobacteriaceae *to thrive. This microbial shift reflects competitive release-where the decline of antibiotic-sensitive *Staphylococcus spp* creates an ecological niche for resistant *Enterobacteriaceae* to dominate. The reduction in *S. aureus* isolates in 2019-2020 coincided with the implementation of stringent anti-COVID measures (e.g., reinforced hygiene protocols, reduced elective invasive procedures) [11]. This underscores *S. aureus’*s susceptibility to infection control practices. However, its resurgence after 2020 (9/37, 24.32% in 2021 and 11/60, 18.33% in 2023) correlates with the relaxation of these measures, reinforcing its role as a nosocomial pathogen associated with surgical site infections and bacteremia. These temporal dynamics highlight the sensitivity of* S. aureus* epidemiology to institutional infection prevention strategies. During the study period, the longitudinal trends in MRSA isolation rates demonstrated relative stability (1-2 isolates per year), punctuated by a sharp peak in 2017 (10/26, 38.46%). This suggests a transient outbreak or a surveillance artifact, such as enhanced screening or temporary changes in laboratory protocols. Further analysis of testing practices during this period would help clarify this observation. Notably, no cases were detected from 2019 to 2022, potentially due to improved infection control, reduced antibiotic pressure, or pandemic-related underreporting. A single isolate was detected in 2023, indicating persistent, low-level circulation. While the overall trend is encouraging, the 2017 surge underscores the need for sustained monitoring to prevent resistance from resurging. Aminoglycoside resistance showed a significant decline over time. In contrast, resistance to fluoroquinolones remained stable (*P* = 0.079).

Our microbiological data challenge the current empirical therapy and prophylactic recommendations for osteoarticular infections. We observed a striking predominance of resistant *Enterobacteriaceae*, including 168/415 (40.5%) AmpC producers, which render AMC (used in our setting) ineffective, and 108/415 (26.02%) ESBL producers. In contrast, *Staphylococcus spp* accounted for only 183/813 (22.51%) of isolates, with *S. aureus* comprising 129/813 (15.86%) of these. The threat is further compounded by the presence of carbapenem-resistant *A. baumannii *(40/71, 56%) and CRE (40/415, 9.64%). These findings support abandoning AMC in settings where AmpC producers are prevalent. In such contexts, piperacillin-tazobactam may serve as an interim option. Ertapenem should be reserved for high-risk ESBL cases and used judiciously, while culture-guided prophylaxis is recommended for patients with a history of MDR infections [26]. However, rising resistance in non-fermenters, such as imipenem-resistant *A. baumannii,* underscores the need for novel protocols. Finally, these findings can inform probabilistic choices for empirical antibiotic therapy, since empiric treatment should be tailored to local epidemiology, patient-specific risk factors, and infection severity, rather than relying solely on international guidelines [26].

Our study has some limitations. In particular, we did not stratify between native joint infections, hematogenous osteomyelitis, and device-related infections. Because these entities often differ in their microbiological profiles, this lack of distinction limits the comparability of our results with other studies. Additionally, the retrospective, single-center design is inherently susceptible to selection and reporting biases, which may affect the generalizability of our findings. However, it is noteworthy that our city has only two university hospitals, including Ibn Tofail University Hospital, where this study was conducted. Therefore, our data likely capture a substantial proportion of complex osteoarticular infections managed at the tertiary-care level in this region, enhancing their local representativeness. By acknowledging these limitations, we aim to facilitate a transparent interpretation of our results and underscore their primary value for local epidemiological surveillance and antimicrobial stewardship.

## Conclusions

Our retrospective longitudinal study conducted in our setting revealed a predominance of *Enterobacteriaceae* in osteoarticular infections, along with a marked rise in antimicrobial resistance. This evolving epidemiology underscores the urgent need to revise existing protocols for both prophylactic and empirical therapy, ensuring that they reflect current resistance patterns and support optimal patient care across all stages of prevention and treatment.
